# Post-exercise Effects and Long-Term Training Adaptations of Hormone Sensitive Lipase Lipolysis Induced by High-Intensity Interval Training in Adipose Tissue of Mice

**DOI:** 10.3389/fphys.2020.535722

**Published:** 2020-11-25

**Authors:** Yang Liu, Gaofang Dong, Xiaobo Zhao, Zerong Huang, Peng Li, Haifeng Zhang

**Affiliations:** ^1^Physical Education College, Hebei Normal University, Shijiazhuang, China; ^2^Provincial Key Lab of Measurement and Evaluation in Human Movement and Bio-Information, Hebei Normal University, Shijiazhuang, China

**Keywords:** high-intensity interval training, catecholamine, long-term adaptations, post-exercise effects, lipolysis, hormone-sensitive triglyceride lipase

## Abstract

Although studies have proven that high-intensity interval training (HIIT) shows a comparable effect to moderate-intensity continuous training (MICT) on reducing body fat, especially visceral fat, the mechanism is still unclear. Since MICT consumes more fat during exercise, the mechanism of HIIT weight loss may be related to post-exercise effects, long-term adaptive changes, and hormone sensitive lipase (HSL). The objective of this study was to compare the post-effects of acute exercise, long-term adaptive changes on HSL activity, and catecholamine-induced lipolysis between HIIT and MICT. Following a 14-week high-fat diet (HFD), obese female C57Bl/6 mice were divided into acute exercise groups (one time training, sacrificed at rest and 0, 1, and 12 h after exercise, *n* = 49), -L groups (12-week long-term training, 12-h fasting, *n* = 21), and -C groups (12-week training, primary adipocytes were isolated and stimulated by catecholamine *in vitro*, *n* = 18). MICT or HIIT treadmill protocols (running distance matched) were carried out during training. Comparison of acute exercise effects by two-way ANOVA showed no time × group interaction effect, however, a significant increase in HSL-Ser563 (at 0 and 1 h) and Ser660 phosphorylation (at 0, 1, and 12 h) in inguinal (subcutaneous) fat was only observed in HIIT mice (*p* < 0.05 vs. rest), but not in MICT mice. The periuterine (visceral) fat HSL expression and phosphorylation of HIIT mice was similar to or lower than MICT mice. After long-term training, 12-h fasting significantly increased periuterine fat Ser563 phosphorylation in HIIT mice (*p* < 0.05), but there was no change in MICT mice. Under stimulation of catecholamine *in vitro*, isolated primary adipocytes from periuterine fat of long-term HIIT mice showed a higher Ser563 increase than that found in MICT mice (*p* < 0.05). The quantity of triglyceride (TG) lipid bonds (representing lipolysis level) was significantly lower after HIIT than MICT (*p* < 0.05). The results indicate that (1) acute HIIT can induce an increase of HSL phosphorylation in subcutaneous fat lasting at least 12 h, implying longer post-exercise lipolysis than MICT and (2) long-time HIIT has a better effect on improving catecholamine resistance of visceral adipocytes caused by a HFD, which allows fat to be mobilized more easily when stimulated.

## Introduction

Obesity is a serious threat to human health and has become an important topic in sports science. Traditionally, moderate-intensity continuous training (MICT) is widely used for reducing body fat because long-duration moderate-intensity exercise increases the total amount of skeletal muscle fat oxidation, while high-intensity exercise can only be maintained by glycogen (Frayn, 2010). However, recent studies suggested that long-term high-intensity interval training (HIIT) may have comparable effects on body fat reduction to MICT (Keating et al., 2017; Viana et al., 2019). Since the absolute value of muscle fat consumption during MICT is higher than during HIIT, it may be a reasonable explanation that the higher fat loss occurs after, but not during, HIIT (Abbasi, 2019). Two potential processes may be involved in this observation: (1) In a recovery period, after each acute exercise session, more fat is consumed due to the post-exercise effect of HIIT (acute effect); or (2) Long-term HIIT can cause adipose tissue adaptive changes, which make it easier to be mobilized and metabolized (sustained effect).

Excessive accumulation of triglycerides (TGs) is a major feature of obesity. TG stored in intracellular lipid droplets of white adipose tissue (WAT) accounts for most of systemic reserves (Braun et al., 2018). The first step of fat decomposition is the hydrolysis of TG (so called lipolysis or fat mobilization) into glycerol and non-esterified fatty acids (NEFA). Lipolysis is important because it is the startup phase for oxidation, gluconeogenesis, and redistribution of fat to skeletal muscle (Thompson et al., 2012; Kuo and Harris, 2016). Lipolysis occurs in a sequential manner from TG, diacylglycerol (DAG), and monoacylglycerol (MAG) to produce three NEFAs and one glycerol. Adipose TG lipase (ATGL) and hormone sensitive lipase (HSL), located on lipid droplet membranes, are key rate-limiting enzymes during this process (Berraondo and Martínez, 2000). The main function of ATGL is to hydrolyze TAG into DAG, while HSL exhibits a wider substrate specificity, which can catalyze the hydrolysis of TAG, DAG, and MAG (Kim et al., 2016). Meanwhile, the lipolysis activity of HSL can be regulated by both extra and intracellular signals such as hormones, sympathetic nerves, and intracellular energy receptors through some site-specific serine phosphorylation (Stallknecht et al., 2001; Thompson et al., 2012; Braun et al., 2018). Wide substrate specificity and integration of extra and intracellular signals suggest that HSL activity can represent lipolysis. Unfortunately, the effects of HIIT on HSL lipolysis activity after exercise, as well as the long-term adaptive changes of adipocyte to HIIT, are still not well-studied.

Catecholamines (Epinephrine, E and Norepinephrine, NE) are well-known as the most important signal to regulate lipolysis and HSL activity (Thompson et al., 2012). Studies showed that lipolysis during exercise mainly depended on the adrenal glands, rather than sympathetic nerves (Stallknecht et al., 2001), and the amount of E and NE secreted by the adrenal glands increased with exercise intensity (McMurray et al., 1987), which is the primary determinant of the plasma catecholamine response (Zouhal et al., 2013). Low-to-medium-intensity exercise can cause a modest increase of circulating catecholamines and HSL lipolysis activity by the β_3_ adrenergic receptor (β_3_AR)-Gs protein pathway. However, acute high-intensity exercise inhibits HSL activity through the α-adrenergic receptor (αAR)-Gi protein pathway (Frayn, 2010), and a restriction in the adipose tissue blood flow also occurs (Hodgetts et al., 1991), due to excessive catecholamine levels. Therefore, although HIIT can cause a higher increase of blood catecholamines (Bracken et al., 2009), MICT causes higher lipolysis than HIIT during exercise (Horowitz, 2003). Studies confirmed that HIIT could cause higher blood catecholamine levels than MICT, as well as take a longer time to return to a resting level of catecholamines after exercise (Williams et al., 2013; Evans et al., 2016; Verboven et al., 2018). However, although some studies have compared HSL protein expression in muscle and adipose tissue between MICT and HIIT (Samaneh and Nikooie, 2016; Sun et al., 2020), to our knowledge, the catecholamine-mediated HSL phosphorylation and lipolysis were not studied profoundly.

Besides the post-exercise effect, long-term training can also improve the lipolytic capacity of adipose tissue (Romijn et al., 1993). Fasting can activate fat mobilization by the catecholamines-β_3_AR pathway to release more NEFA for energy balance. Obesity and high-fat diets (HFDs) can make adipocytes resistant to catecholamines, which makes HSL lipolysis activation more difficult (Gaidhu et al., 2010); however, long-term aerobic training can improve this resistance to catecholamines (Moro et al., 2009). Endurance training can improve the sensitivity of adipocytes to catecholamines and increase HSL phosphorylation in rodents (Snook et al., 2017) and humans (Bertholdt et al., 2018). The mechanism of HIIT reducing fat is often considered to be related to the recovery period after exercise. However, no study had compared whether HIIT can better relieve the catecholamines resistance of adipocytes and improve the fat mobilization ability when compared to MICT.

In summary, the mechanism of a better weight loss effect with HIIT, when compared to MICT, may be related to the higher level of post-exercise HSL lipolysis after single acute training. It may also be related to long-term HIIT improving the sensitivity of adipocytes to catecholamines, which enhances the lipolysis of HSL. Therefore, based on the two potential processes about acute and sustained effect, the purpose of this study was to test two hypotheses: (1) acute HIIT induces stronger HSL phosphorylation than MICT after exercise and (2) long-term HIIT improves the catecholamines resistance of adipocytes caused by HFD, and increases HSL phosphorylation and lipolysis more than MICT. In this study, treadmill training animal model was used to compare the effects of HIIT and MICT on HSL phosphorylation and lipolysis in adipose tissue. These results would provide new evidences to explain the underlying mechanisms about HIIT fat loss effect.

## Materials and Methods

### Study Design

**Figure 1:**
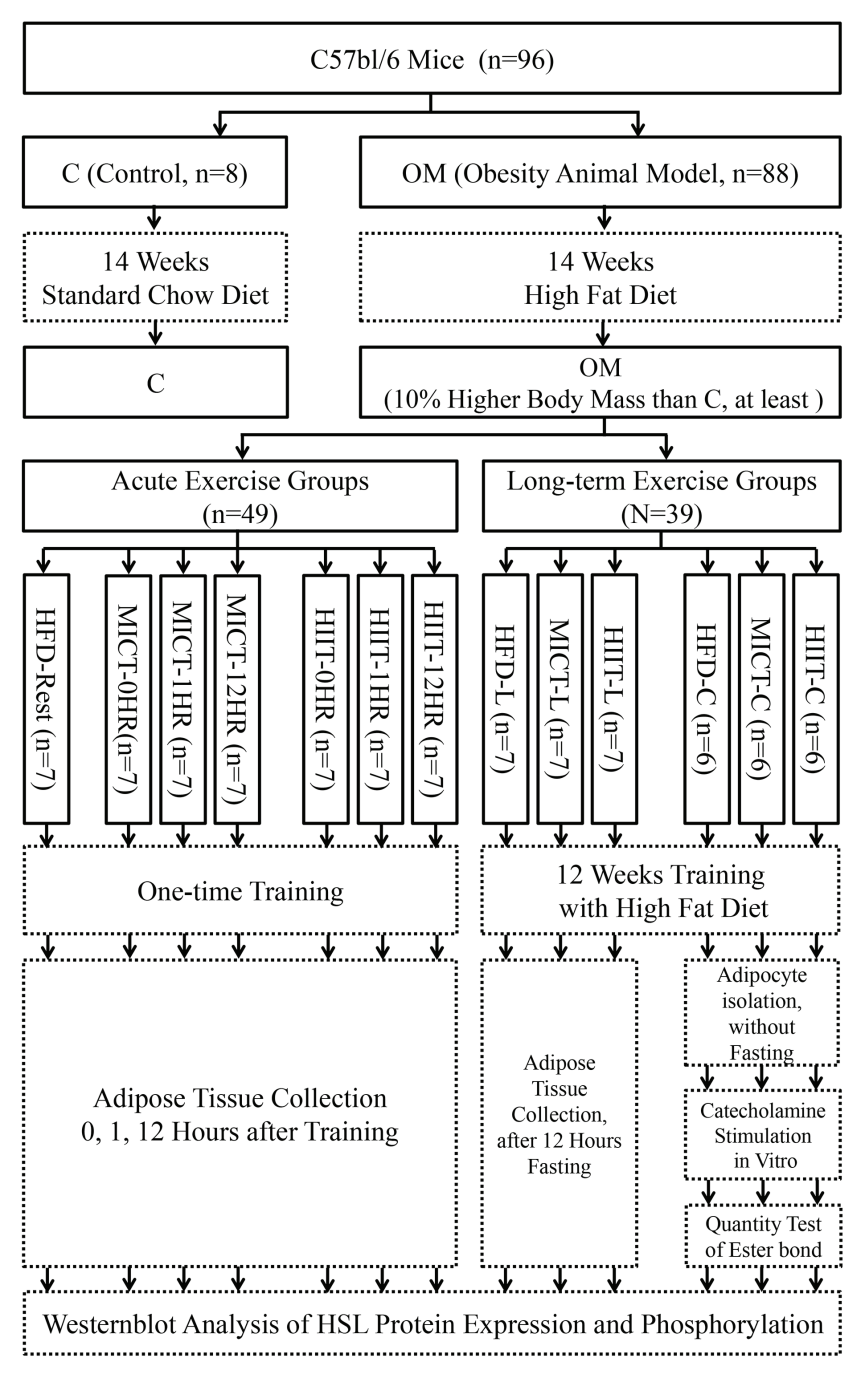
Study design. Experimental groups are shown in solid boxes. Contents of intervention and test are shown in dotted boxes. The suffix “-nHR” means “n hours after acute exercise groups,” “-L” means “long-term training groups,” and “-C” means “primary adipocyte isolation groups after long-term training.”

The study design was shown in Figure 1. Since it is well-known that the distribution of α and β_3_ARs, which receive catecholamine lipolysis signal, is not exactly same between sexes (Zouhal et al., 2013), and most of our previous HIIT human studies involved young women (Zhang et al., 2017, 2020), for maintaining research continuity, female C57Bl/6 mice (4 weeks old, Vital River Laboratories) were selected as animal models. Mice were housed, one per cage, under controlled temperature conditions (20–24°C) with a 12 h light/ dark cycle. The control group (C, *n* = 8) was randomly selected and fed standard mice chow, while the remaining mice (as obesity model group, OM) were fed a HFD (60% standard chow, 16% sugar, 5% fat, 18% egg yolk powder, and 1% sodium cholate). The control group was set up as the basis for comparison to determine the effect of a HFD. After 14 weeks, when the body mass of every mouse was at least 10% higher than the mean value of the C group, mice in the OM group were considered to be established as obese animal models. Three separate experiments were executed: (1) An acute exercise experiment to observe post-exercise changes in HSL activity. Forty-nine OM mice were randomly divided into control (HFD-Rest, *n* = 7), MICT (-0, -1, and -12HR, *n* = 7 each), and HIIT (-0, -1, -12HR, *n* = 7 each) groups, and each group was subjected to one-time exercise; (2) A long-term exercise experiment to observe the adipocyte adaptive changes of HSL activation. Twenty-one OM mice were randomly divided into 3 -L groups (HFD-L, MICT-L, and HIIT-L, *n* = 7 each), which were subjected to long-term exercise. HSL expression and phosphorylation were tested after 12 h of fasting; (3) An *in vitro* catecholamines stimulation experiment to observe catecholamine-induced lipolysis and HSL activation of adipocytes after long-term exercise. Eighteen OM mice were randomly divided into 3 -C groups (HFD-C, MICT-C, and HIIT-C, *n* = 6 each) for primary adipocyte isolation, and each were subjected to long-term exercise. The quantity of triglyceride bonds and HSL were tested after catecholamines stimulation. The intake of food and water was free, and no statistical difference was found between HFD groups during the study period. Random numbers generated by Excel 14.0 were used to perform the randomizations. All experimental procedures were approved by the Ethics Committee of Hebei Normal University.

### Exercise Protocol

First, MICT and HIIT mice were adapted to a treadmill for 3 days (10 min/day, 5 m/min, 0°). Training rodents were subjected to a 25° uphill treadmill training once (acute exercise groups) or for 12 weeks (-L and -C groups), while HFD mice rested as a control. HIIT consisted of a series of running for 1 min at maximum speed followed by 1 min of running at medium speed (1–1 min cycle). Running speeds were determined according to an incremental exercise test (IET), based on previous research (Høydal et al., 2007; Pereira et al., 2012; Dehghani et al., 2018). The initial velocity of IET was 6 m/min, after which it increased by 3 m/min for 3 min until exhaustion, which was defined when mice could not keep up with the velocity five times in 1 min despite being physical poked. The maximum running velocity of HIIT equaled the exhaustion speed (ES) of last stage in IET. The interval medium speed of HIIT and the continuous speed of MICT both equaled 55% of ES. One MICT training lasted for 45 min. In order to match the exercise volume, the number of 1–1 min cycles in each HIIT was calculated based on the speeds of MICT to ensure equal running distance. Mice of long-term groups were trained 5 days continuously and given 2 days rest in each week. An IET was conducted every 2 weeks to ensure that running speed would accommodate growth. The running speeds of MICT increased from 13 to 17 m/min and maximum running speeds of HIIT increased from 23 to 31 m/min throughout the training period.

### Sample Collection

All mice were sacrificed after anesthesia (pentobarbital sodium, Solarbio, I.P.). Mice of the -0, -1, and -12HR groups were killed at 0, 1, or 12 h, respectively, after one-time training to determine the peak or recovery period of catecholamines secretion (Williams et al., 2013; Sgrò et al., 2014). After training ended at 12 weeks, mice of -L groups would rest for 48 h, fast for 12 h, and be killed successively. Mice of -C groups would rest for 60 h without fasting and be killed. The inguinal and periuterine (around the uterus) fat pads were used to represent subcutaneous WAT (SCAT) and visceral WAT (VAT). Gastrocnemius samples of -L groups were collected to represent skeletal muscles. Briefly, fat pads and muscle samples were extracted, and fat pads were weighed. Five samples of each -L group were randomly selected for H&E staining. Center pieces were cut-off and fixed with 4% paraformaldehyde to avoid being torn. Rest tissues of -L group and the remaining fat pad tissues from acute exercise groups were frozen in liquid nitrogen and stored in the −80°C refrigerator (DW-86, Haier) for western blot analysis and a muscle TG content test. Samples of -C group were minced and put into tubes containing phosphate-buffered saline (PBS, Solarbio) for primary adipocyte isolation.

### Histological Observation of Adipose Tissue

The volume of adipocytes was observed by H&E staining. Briefly, tissues were fixed with 4% paraformaldehyde (Solarbio) for 48 h, embedded in paraffin, and cut into 5 μm sections. After xylene dewaxing and alcohol rehydrating, samples were subsequently stained with hematoxylin (Solarbio) for 5 min, washed with water and 0.6% ammonia, stained with eosin (Solarbio) for 2 min, and dehydrated with alcohol. Finally, sections were sealed with resin (Solarbio) and photographed using an optical microscope. At least, 150 adipocytes per group were analyzed for cell surface areas by ImageJ 1.51, and the investigator was blinded with regard to group allocation. Overall results were represented by representative images.

### Primary Adipocyte Isolation and Catecholamine-Induced Lipolysis Test *in vitro*

To observe the adaptive changes after long-term training, we tested the catecholamine-mediated lipolysis ability of adipocytes. NE was selected to represent catecholamines for stimulating adipocyte, since it can be secreted by both sympathetic nerves and adrenal glands. After adipocytes were isolated from fat pads and stimulated by physiological concentrations of NE *in vitro*, the quantity of triglyceride bonds (ester bond, “-O-CO-”), which could be hydrolyzed by HSL, was determined by Fourier Transform Infrared (FTIR) spectroscopy. Each triglyceride molecule contains three triglyceride bonds. The physiological function of HSL is to hydrolyze triglyceride bonds to carboxyl (“-COOH”) and hydroxyl (“-OH”) and it can convert triglyceride into glycerol and fatty acids; therefore, the reduction of triglyceride bonds represents the ability of HSL to hydrolyze TG bonds. Adipocyte isolation and epinephrine stimulation were carried out according to Gaidhu et al. (2010) and followed by FTIR spectroscopy (Kucuk Baloglu et al., 2017). Briefly, tissues were digested with collagenase (Solarbio) for 30 min at 37°C with gentle agitation, centrifuged (500 *g*, 5 min), washed by collagenase-free PBS (three times), and filtered through a nylon mesh (Jingan Bio). Adipocytes were washed off, collected, and equilibrated for 30 min before the experiment. Isolated cells were transferred to a medium containing fetal bovine serum (10%, Tianhang Biotechnology) and epinephrine hydrochloride (100 nM, Sigma) at 37°C for 75 min. After epinephrine stimulation, the cells were collected, lysed by RIPA (Solarbio), and centrifuged. The lipid layer above the solution was extracted using chloroform (1:1.5). Extraction solutions were mixed with anhydrous sodium sulfate (1:0.05) and allowed to stand for 2 h for dehydration. Samples were scanned on a FTIR spectrometer (Vertex 70, Bruker) over the 370–4,000 cm^−1^ spectral range. A peak near 1,740 cm^−1^ represented the amount of triglyceride bonds present. The area under the curve (AUC) in the range of 1,710–1,770 cm^−1^ was calculated to quantify the presence of triglyceride bonds. Spectral curve drawing and ACU calculation were performed using OriginPro 8 (Origin Lab Corporation). For quality control, three randomly selected samples were measured three times and the obtained Intraclass correlation coefficient was 0.98 (good reliability).

### Western Blot Analysis for Adipose Tissue

In order to observe the post-exercise activity and long-term exercise induced adaptation of HSL, protein expression and phosphorylation of VAT, SCAT, and isolated adipocytes were tested after epinephrine stimulation (see details in *in vitro* Lipolysis Test part). Chopped tissue and isolated cells were homogenized with RIPA buffer (Solarbio) supplemented with protease and phosphatase inhibitor (Thermo Fisher Scientific). After the homogenate was centrifuged (4°C, 14,000 *g*, 10 min), the lower layer was extracted and boiled at 98°C for 5 min. Samples were electrophoresed in 12% SDS-PAGE gel and transferred onto polyvinylidene difluoride membranes (Millipore). Primary antibodies were used to detect HSL, HSL-Ser563, and HSL-Ser660 (#4107, #4139, and #4126, Cell Signaling Technology, all by dilution of 1:2,000). Goat anti-rabbit IgG secondary antibody (GB23303, Servicebio, by dilution of 1:5,000) was used for luminescence. Actin (primary antibody No. AP0060, Bioworld Tech, by dilution of 1:5,000) was used as the loading control. The ECL (Solarbio) excited luminescence was collected and analyzed by the gel imaging system (Fusion Fx5-xt, VILBER LOURMAT). Overall results were represented by representative bands.

### Test of Skeletal Muscle TG Content

Triglyceride content of skeletal muscle was tested using the GPO-PAP enzyme method. Chopped muscle samples were homogenized with RIPA buffer (Solarbio) and tested using TG kits (Prod No. A110-1-1, Jiancheng Bioengineering Institute). For quality control, seven randomly selected samples were measured two times and the obtained Intraclass correlation coefficient was 0.77 (good reliability).

### Statistical Analyses

Data are presented as means ± SD. Differences in body mass, fat pad weight, muscle TG, spectral AUC, HSL protein expression, and phosphorylation of -L group were analyzed using a one-way ANOVA. Differences of HSL in acute exercise groups were analyzed with a two-way ANOVA (time and training type as two factors). Main effect and least significant difference (LSD) *post hoc* tests were used to compare treatments when no significant interaction was found. Statistical significance was set at *p* < 0.05. According to the data of fat pad mass in previous experiments, the minimum sample size is 4 (*α* = 0.05, power = 0.8).

## Results

### HSL Protein Expression and Phosphorylation 0–12 h After Acute Training

**Figure 2:**
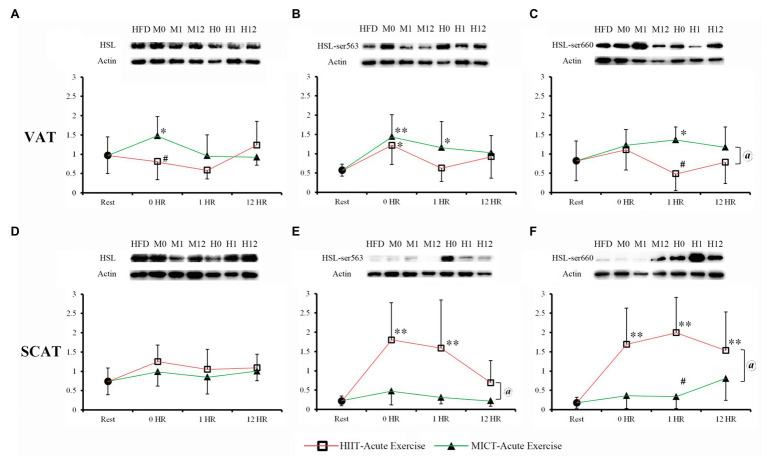
Hormone sensitive lipase (HSL) protein expression and phosphorylation of visceral WAT (VAT; **A**-**C**) and SACT **(D-F)** after one-time acute exercise. High-fat diet (HFD) or rest: HFD-Rest group, set as baseline without training; M0, M1, and M12: moderate-intensity continuous training (MICT)-0HR, -1HR, and -12HR; H0, H1, and H2: high-intensity interval training (HIIT)-0HR, -1HR, and -12HR; ^*^p < 0.05 vs. rest; ^**^p < 0.01 vs. rest; ^#^*p* < 0.05 HIIT vs. MICT; and ^@^Main effect of the training type (MICT vs. HIIT) *p* < 0.05, when no significant interaction between two factors (training type and time point) was found. Values of *p* between HFD-Rest and acute exercise groups were calculated by one-way ANOVA, and values of *p* between MICT and HIIT were calculated by 2 × 3 two-way ANOVA. Bands shown are representative for overall results.

To compare the post-exercise effects of MICT and HIIT on lipolysis activity of HSL, we observed the protein expression and phosphorylation of Ser563 and Ser660 at 0, 1, and 12 h after one-time acute exercise (Figures 2A–F). Except for the significant increase of VAT in MICT-0HR group, HSL expressions at 0–12 h after acute exercise were not significantly different from sedentary mice (HFD-Rest), regardless of VAT or SCAT (Figures 2A,D). VAT ser563 of MICT-0HR, HIIT-0HR, and MICT-1HR significantly increased when compared to HFD-Rest. MICT groups overall had significantly more VAT ser660 than HIIT, while only MICT-0HR had significantly more compared to the resting group. Unlike VAT, HSL phosphorylation, both Ser563 and Ser660, of SCAT in HIIT groups was significantly higher than that found in MICT groups, while no difference was found between MICT groups and the resting group, with the exception of MICT-12HR for Ser660. For Ser563 and Ser660, HSL phosphorylation in HIIT groups was significantly higher than the resting group, except at 12 h for Ser563. To summarize, in SCAT, HSL phosphorylation in HIIT mice was higher than in MICT mice, but in VAT, the phosphorylation in HIIT mice was lower than in MICT mice at 1 h after exercise.

### Changes in Body Mass, Fat Pad Weight, and Adipocyte Morphology After 12 Weeks of Training

To compare the effect of catecholamines resistance by HIIT and MICT, OM mice were first fed a HFD for 14 weeks to establish obese animal models. After 14 weeks, the average body mass of OM was 20% higher than the control group (standard chow diet), even the lightest mouse of OM was 12% higher (Figure 2A). To compare the weight loss effects of HIIT and MICT, 21 mice were randomly selected from the OM group and divided into HFD-L, MICT-L, and HIIT-L for 12 weeks of training (Figure 1). No body mass difference between three groups before training was found (Figure 2A).

**Figure 3:**
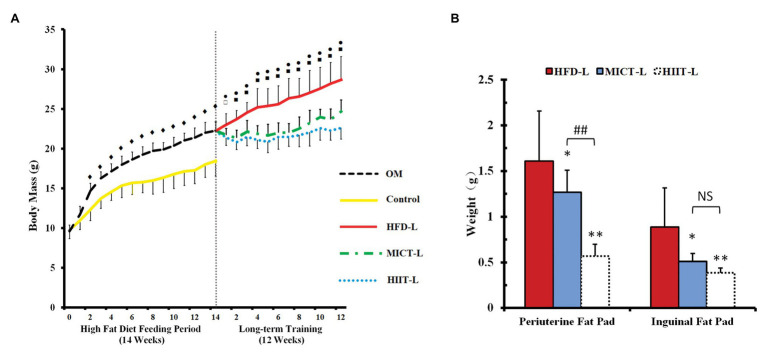
Body mass **(A)** and fat pads weights **(B)** of long-term exercise groups. Periuterine fat pad represents visceral fat, while inguinal pad represents subcutaneous fat. Data are mean ± SD. **(A)** ^♦^*p* < 0.01 OM vs. C, ^⚫^*p* < 0.01 HIIT-L vs. HFD-L, ^▫^*p* < 0.05 MICT-L vs. HFD-L, ^▪^*p* < 0.01 MICT-L vs. HFD-L, and no significant difference of body mass between MICT-L and HIIT-L. **(B)** ^*^p < 0.05 vs. HFD-L, ^**^p < 0.01 vs. HFD-L, ^##^*p* < 0.01 MICT-L vs. HIIT-L, and NS, no significant difference.

**Figure 4:**
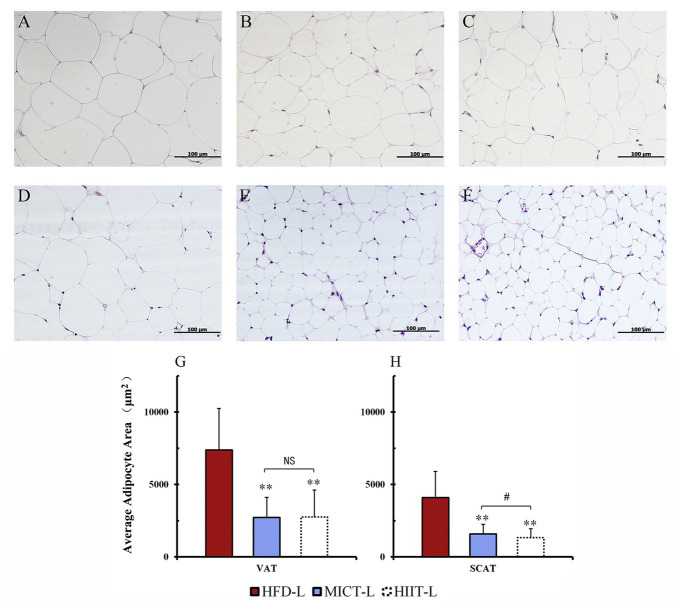
Adipocyte sizes of different fat depots. Periuterine adipocytes (VAT) of HFD-L **(A)**, MICT-L **(B)**, and HIIT-L **(C)**, as well as inguinal adipocytes (SCAT) of HFD-L **(D)**, MICT-L **(E)**, and HIIT-L **(F)** were shown by H&E staining; Average adipocyte area **(G,H)**; ^**^p < 0.01 vs. HFD-L, ^#^*p* < 0.05 MICT-L vs. HIIT-L, and NS, no significant difference. Images shown are representative for overall results.

After 12 weeks of exercise, the average body mass of HIIT-L and MICT-L was 22 and 15% lower than HFD-L (sedentary, both *p* < 0.01), respectively, while no significant difference was found between HIIT-L and MICT-L (Figure 3A). Inguinal fat weights of HIIT-L and MICT-L, as well as periuterine fat weights, were significantly lower than HFD-L (*p* < 0.05 or *p* < 0.01, Figure 3B). Periuterine (but not inguinal) fat weights of HIIT-L were lower than MICT-L (*p* < 0.01, Figure 3B). H&E staining showed that both visceral and subcutaneous adipocyte sizes of exercise groups were lower than HFD-L and SCAT adipocyte sizes of HIIT-L were lower than MICT-L (*p* < 0.05 or *p* < 0.01, Figures 4A–H). These results suggest that long-term training protocols can reduce body mass, fat weight, and adipocyte sizes, and HIIT showed stronger visceral fat mass reducing effect compared to MICT.

### Skeletal Muscle TG Content

**Figure 5:**
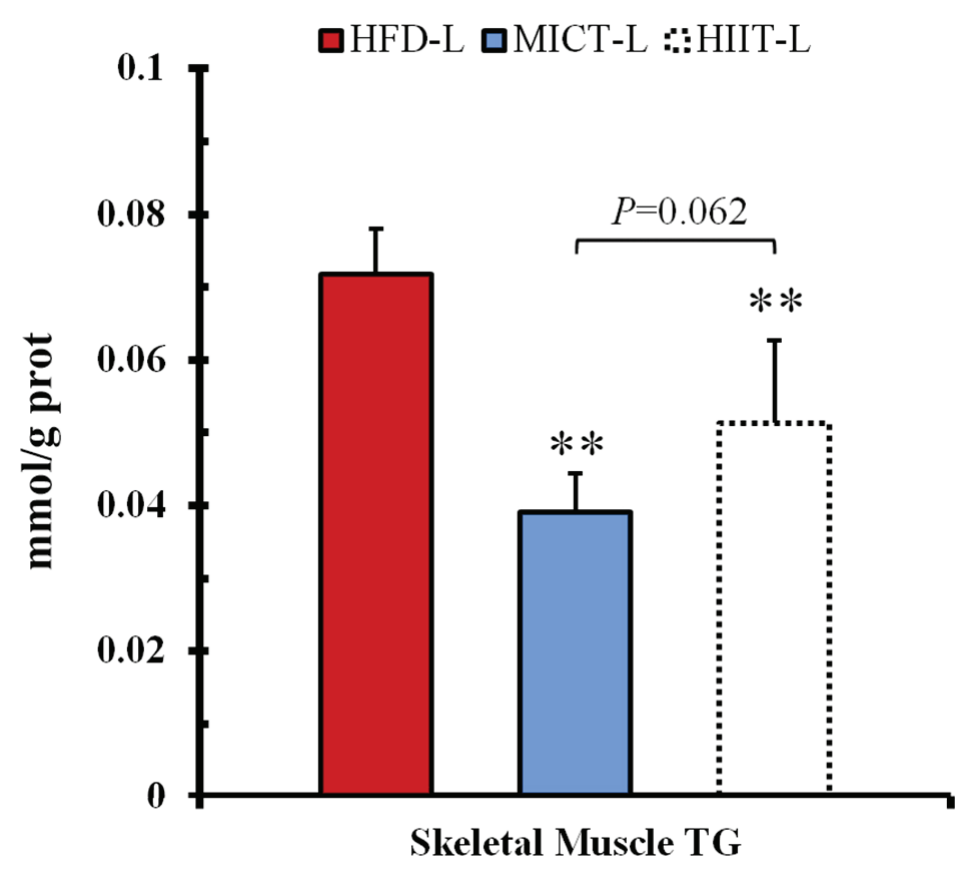
Skeletal muscle triglyceride (TG) content. ^**^p < 0.01 vs. HFD-L. Values of *p* were calculated by one-way ANOVA.

To test if weight loss with high-intensity exercise is due to the redistribution of hydrocarbon sources between fat and skeletal muscle, we tried to examine the TG content increase of skeletal muscle and decrease of fat pad weight in the -L group. After long-term training, TG of MICT-L and HIIT-L was significantly lower than HFD-L. HIIT-L showed a tiny higher trend than MICT-L, but without significant difference (*p* = 0.062, see Figure 5).

### HSL Protein Expression and Phosphorylation During Fasting After 12 Weeks of Training

**Figure 6:**
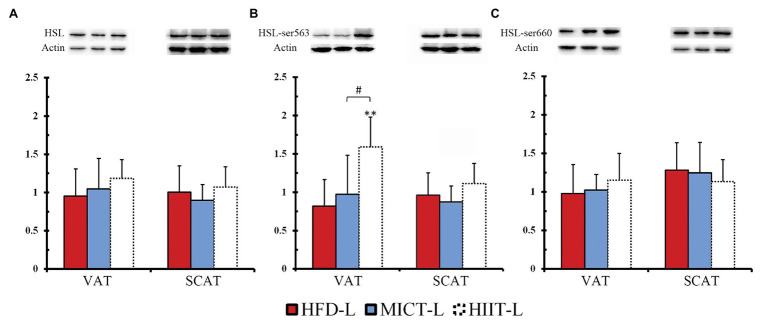
HSL protein expression **(A)**, phosphorylation of ser563 **(B)** and ser660 **(C)** with 12 hours of fasting and after 12 weeks of MICT or HIIT. ^**^p < 0.01 vs. HFD-L; ^#^*p* < 0.05 HIIT-L vs. MICT-L. Values of *p* were calculated by one-way ANOVA. Bands shown are representative for overall results.

To compare the effects of long-term HIIT and MICT on fat mobilization, we observed HSL protein expression and phosphorylation of -L groups during fasting (Figures 6A–C). In VAT, after 48 h resting and 12 h fasting, Ser563 phosphorylation of HIIT-L was significantly higher than HFD-L and MICT-L (Figure 6B), even though HSL expression and Ser660 phosphorylation were similar among these groups. No significant differences were found in SCAT. This suggests that with the same hunger stimulation, HSL-Ser563 of VAT adipocytes can be phosphorylated easier after long-term HIIT.

### HSL Phosphorylation and Triglycerides Bonds Quantity of Isolated Adipocytes After Incubation With Epinephrine

**Figure 7:**
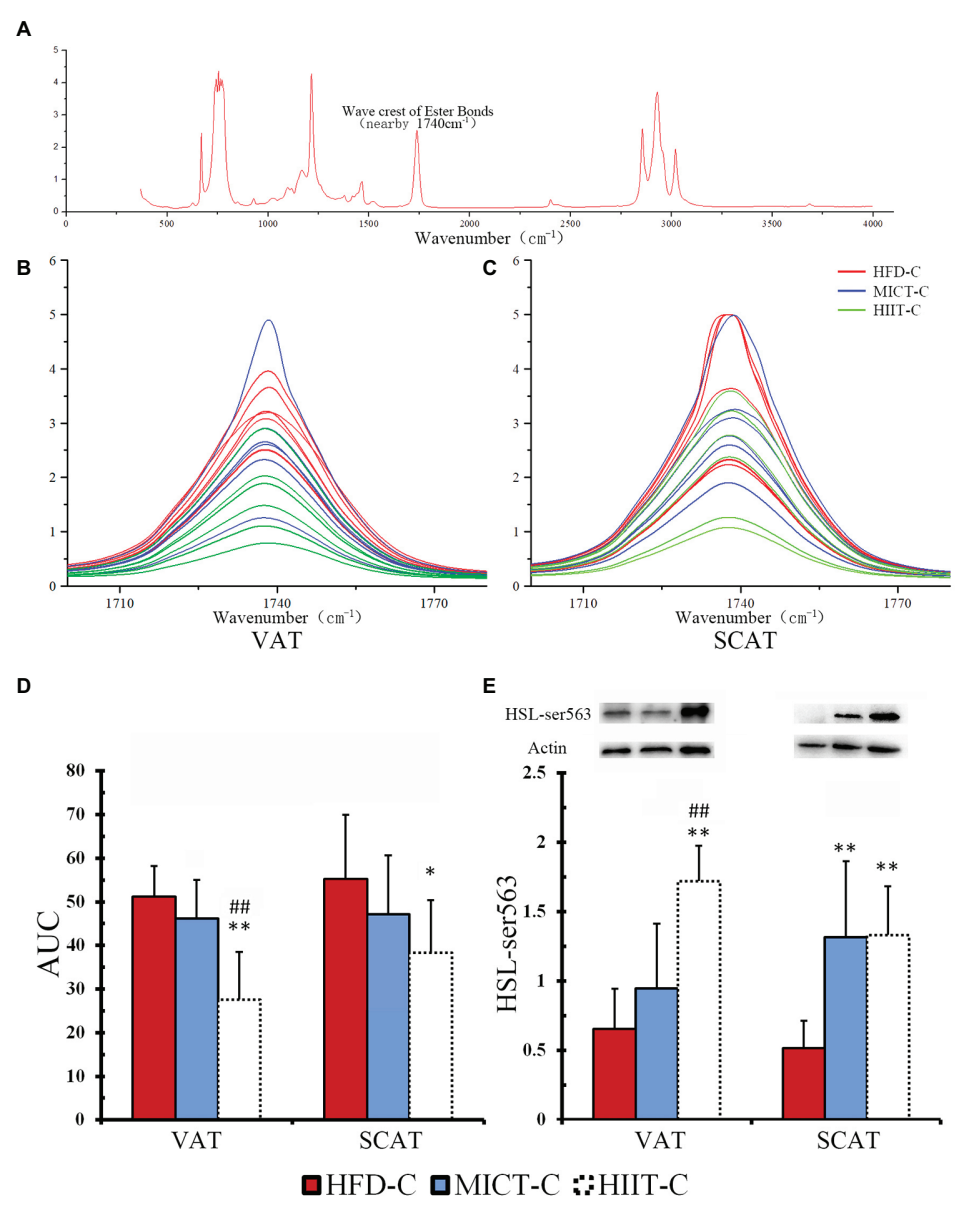
A complete FTIR spectrum **(A)** is shown, including a peak nearby 1,740 cm^−1^ representing the quantity of triglyceride bonds. Peaks of VAT **(B)** and SCAT **(C)** are overlapped to show the difference between HFD-C, MICT-C, and HIIT-C. The area under the curve (AUC), representing the quantity of triglyceride bonds. Differences are shown in the amount of triglyceride bonds **(D)** and HSL-Ser563 phosphorylation **(E)** between HFD-C, MICT-C, and HIIT-C after physiological concentration of epinephrine stimulation (simulated fasting *in vitro*). ^*^p < 0.05 vs. HFD-C; ^**^p < 0.01 vs. HFD-C; and ^##^*p* < 0.01 HIIT-C vs. MICT-C. Bands shown are representative for overall results.

To determine if long-term HIIT is more effective than MICT at improving HSL-mediated fat mobilization, we compared Ser563 and triglyceride bonds of adipocytes with epinephrine stimulation (to imitate fasting *in vitro*). We found that fasting-induced HSL-Ser563 phosphorylation of VAT significantly more in the HIIT-L group. Figure 7A shows a complete FTIR spectrum, including a peak near 1,740 cm^−1^ representing the quantity of triglyceride bonds. Peaks of all triglyceride bonds are overlapped and most peak heights of HIIT-C are lower than the average height of HFD-C and MICT-C, in both VAT and SCAT (Figures 7B,C). ANOVA of AUC showed both VAT and SCAT of HIIT-C were significantly lower than HFD-C (*p* < 0.05 or *p* < 0.01, Figures 7D,E), and VAT of HIIT-C was also lower than MICT-C (*p* < 0.01, Figure 7D). Ser563 of HIIT-C was significantly higher than in HFD-C and MICT-C and was similar to changes after fasting in VAT. In SCAT, both Ser563 of MICT-C and HIIT-C were higher than in HFD-C, although they were not significantly different (Figure 7E).

## Discussion

This study compared HIIT and MICT for acute and sustained effects involved in the regulation of weight loss and lipolysis. The major findings of this study were that: (1) one-time acute HIIT can increase HSL lipolysis activity of SCAT more than MICT after exercise and (2) compared with MICT, long-term HIIT can increase visceral adipocytes lipolysis sensitivity to catecholamines, and increase HSL-Ser563 phosphorylation of VAT during fasting or catecholamines stimulation. Incidentally, we found that the TG content of skeletal muscle in HIIT mice showed tiny higher trend (*p* = 0.062) than that of MICT mice after long-term training, but not lower. It suggested that increased lipolysis caused by HIIT during a recovery period or fasting may cause redistribution of TG from adipose tissue to muscle for increasing energy substrate storage, which may be a kind of “training paradox” (Russell, 2004). However, since no statistical difference was found, future studies are needed to explore this phenomenon.

Previous studies focused on comparing the protein expression of HSL in muscle or adipose tissue between MICT and HIIT (Samaneh and Nikooie, 2016; Sun et al., 2020), and very few studies have compared the HSL phosphorylation and lipolysis during fasting or after catecholamine stimulating. Our findings may explain why HIIT has comparable subcutaneous and visceral fat reducing effects, even though the fat oxidation rate of high-intensity training is lower than moderate-intensity training during exercise.

### HIIT Has Similar Body Mass Controlling Effects and Better Visceral Fat Reduction Effects Than MICT

From the perspective of the energy substrates proportion, MICT has the highest fat consumption rate during exercise. However, some human studies have shown that HIIT can achieve a similar fat loss effect than MICT and be more time efficient (Keating et al., 2017; Viana et al., 2019). Since visceral fat accumulation is a risk factor for a number of metabolic diseases, researchers have also observed the effectiveness of HIIT and MICT in reducing visceral fat in human trials. Some human studies found that HIIT reduced visceral fat similarly to MICT (Zhang et al., 2017; Tong et al., 2018), while others showed that HIIT can reduce more visceral fat (Maillard et al., 2016; Zhang et al., 2020). Animal studies have shown similar results. Wang et al. (2017) showed that of mice fed a HFD, the HIIT group had a lower body fat percentage than the MICT group. Similarly, it has been shown that HIIT could significantly reduce the abdominal and epididymal fat mass in Zucker rats (a genetic model of obesity) and HFD-fed SD rats, when compared to MICT (Kapravelou et al., 2015; Shen et al., 2015; Maillard et al., 2019). The present results showed similar results to some previous studies. In 12 weeks of training, HIIT and MICT mice lost a similar amount of body mass and inguinal fat weight; however, the periuterine fat weights of HIIT groups were lower than MICT groups. This data indicate that long-term HIIT has a similar effect on controlling fat accumulation as MICT and could reduce more visceral fat.

The mechanisms, by which HIIT can reduce body fat equally and visceral fat mass more efficiently than MICT, are still unclear. This study focused on HSL, a key enzyme for fat mobilization and catecholamine-induced lipolysis. Here, we explain this mechanism with the post-effect of acute exercise and adaptive changes of long-term training of fat mobilization caused by HIIT.

### The Post-exercise Effect of HIIT and MICT: More HSL Lipolysis Activation of Subcutaneous Fat 0–12 h After Acute Exercise

The mechanism of HIIT reducing fat is often considered to be related to the recovery period after exercise (Gentil et al., 2020). A frequently proposed viewpoint is that HIIT can cause higher excess post-exercise oxygen consumption (EPOC) during a recovery period, which makes the total energy expenditure higher than during MICT. Some studies support this view (Malatesta et al., 2009), while others found HIIT caused EPOC similar to that of MICT (Moniz et al., 2019). Another viewpoint is that because HIIT consumes large amounts of glycogen during exercise, fat is oxidized during the recovery period to resynthesize glycogen (Kiens and Richter, 1998). The third viewpoint is that TGs are redistributed from adipose tissue to muscle because of the stronger stimulation of muscle with HIIT, a process related to the “endurance paradox” (Kuo and Harris, 2016). Although the mechanisms underlying these three viewpoints are different, either the increase of EPOC, synthesis of glycogen, or the redistribution of TG requires higher fat mobilization to provide sufficient NEFA. This study attempted to test the regulation of post-exercise HSL lipolysis and expected to provide helpful evidence for the above viewpoints.

Due to the wide substrate specificity and integration of extra and intracellular signals, the activity of HSL could be regarded as a representative of fat mobilization. HSL can be activated by many signals to catalyze the hydrolysis of TAG, DAG, and MAG, through site-specific serine phosphorylation of Ser563, Ser565, and Ser660 (Kim et al., 2016). Firstly, lipolytic hormones transported through blood, such as catecholamines, growth hormone (GH), and atrial natriuretic peptide (ANP) can bind to the membrane receptors of adipocytes and activate the cAMP-PKA or cGMP-PKG pathways and phosphorylate Ser563 and Ser660 to increase the lipolysis activity of HSL. Secondly, sympathetic nerve endings in adipose tissue can also secrete NE and promote lipolysis of HSL. Thirdly, AMPK, an important intracellular energy receptor, can inhibit HSL activity through Ser660 phosphorylation (Stallknecht et al., 2001; Thompson et al., 2012; Braun et al., 2018). It is reasonable to assume that HIIT can also cause a greater post-effect increase in HSL-Ser563 and Ser660 phosphorylation and lipolysis, since intensity is a more important factor than duration of altering catecholamines responses to exercise (Zouhal et al., 2008, 2013). HIIT had been shown to cause high levels of catecholamines secretion within 1–3 h after acute exercise (Williams et al., 2013; Evans et al., 2016; Verboven et al., 2018) and the elevation of catecholamines caused by MICT can return to a resting level within 0–1 h, while the increase caused by HIIT did not recover for 1–3 h (Williams et al., 2013; Sgrò et al., 2014). In our study, HSL expression and phosphorylation were recovered to resting level after 12 h in both MICT and HIIT group, except SCAT Ser660 phosphorylation of HIIT mice. Two-way ANOVA analysis showed that there was no significant interaction effect between training type and time, indicating that the post-exercise change trends of HSL expression and phosphorylation in HIIT groups and MICT groups were similar over time. However, the results of VAT ser660, SCAT ser563, and SACT ser660 (Figures 2C,E,F) showed significant main effects of training types, indicating that HIIT and MICT may have different effects on post-exercise HSL phosphorylation except VAT ser563.

Because visceral fat is more sensitive to catecholamines than subcutaneous fat (Davies et al., 1974), we also assumed that highest HSL phosphorylation after acute exercise should be observed in visceral fat of HIIT mice. Unexpectedly, at 0–1 h after exercise, MICT increased HSL expression and phosphorylation of both Ser563 and Ser660 of VAT, while HIIT did not. At 0–1 h after exercise, HIIT increased HSL phosphorylation of SCAT (but not VAT) by 5–10-fold, while MICT did not. Even after 12 h, the SCAT Ser660 phosphorylation of HIIT mice was still higher than resting level. This indicated that MICT might activate HSL lipolysis of VAT during exercise (Figure 2A), which lasted more than 1 h after acute exercise (Figure 2C), while HIIT may have longer post-effect on HSL lipolysis of SVAT (Figures 2E,F).

Our study showed that MICT can activate HSL lipolysis 0–1 h after exercise and this was consistent with previous studies (Watt et al., 2006; Ogasawara et al., 2010); however, few studies have compared the post-effects of HIIT and MICT. Unexpectedly, HIIT activated HSL of SCAT, but not VAT, which was more sensitive to catecholamines. The inhibition of αAR probably played a key role during this process. It should be noted that the regulation of lipolysis by blood catecholamine concentration has two sides: low-to-medium-intensity exercise can cause a modest increase in catecholamines, which activates HSL phosphorylation and lipolysis through the β_3_AR-Gs protein pathway. The lipolysis rate reaches the maximum at about 65% VO_2_max (Horowitz, 2003). However, when the catecholamines concentration is too high, the αAR-Gi protein pathway is activated, thereby inhibiting HSL phosphorylation and lipolysis (Frayn, 2010). During the first hour after exercise, HIIT caused excessively high concentrations of catecholamines, which inhibited HSL of VAT by αAR; however, because the sensitivity of SCAT was lower, high concentrations of catecholamines caused by HIIT did not activate αAR of SCAT, and still activated β_3_AR and caused a post-exercise increase of HSL lipolysis.

Another problem is that the high blood catecholamines concentration caused by acute HIIT can only last for 1–3 h. It is unclear why HSL-Ser660 of SCAT in the HIIT group remains 5-fold higher than the resting level at 12 h after acute exercise. Another study had found that the level of phosphorylation in HSL was still very high 3 h after MICT (Ogasawara et al., 2010), even though the catecholamines concentration had recovered at this time point. Some studies have also shown that lipolysis continues to increase 24 h after acute exercise (Magkos et al., 2009). These results indicate that the blood catecholamines concentration was not the only regulatory signal for post-exercise HSL lipolysis. Lipolysis activity of HSL is also regulated by other signals such as sympathetic nerves and natriuretic peptides (Braun et al., 2018), and the increase in Ser660 at 12 h after acute HIIT may due to these long-lasting signals.

In summary, HIIT can induce stronger post-exercise HSL phosphorylation in SCAT, which may explain why HIIT and MICT have similar effect on reducing subcutaneous fat. However, the post-effect of HSL on VAT by HIIT is weak and could not explain the phenomenon that HIIT can reduce more visceral fat than MICT.

### Adaptation Changes to Long-Term Training: HIIT Can Improve the Catecholamines Resistance of Adipocytes Caused by a HFD and Increase HSL Lipolysis Activity

Another hypothesis that might explain the fat loss by HIIT, besides acute effects after exercise, are the adaptive changes of adipose tissues caused by long-term training (sustained effects). A HFD can cause catecholamines resistance in adipocytes, which makes lipolysis more difficult. As a result, it is difficult to hydrolyze TG in adipocytes to glycerol and NEFA during fasting or exercise. Gaidhu et al. (2010) found that, although the cAMP-PKA pathway was not affected in mice fed a HFD for 8 weeks, HSL was difficult to be phosphorylated under physiological catecholamines concentrations, indicating that a HFD reduced the sensitivity of adrenergic receptors of adipocytes to catecholamines. Exercise intervention studies have shown that MICT can improve this resistance and increase HSL activity of both humans and rodents (Zouhal et al., 2008; Snook et al., 2017; Bertholdt et al., 2018), and compared with MICT, long-term HIIT could increase HSL expression higher in visceral fat of aged rats (Sun et al., 2020). However, there are few studies comparing the differences between HIIT and MICT in improving catecholamines resistance.

We assumed that long-term HIIT improved catecholamines resistance better than MICT. When mice in the HIIT group were exercising or starving, adipocytes could release more NEFA under catecholamines stimulating, and the body could use more NEFA instead of glucose to maintain energy balance. The data from the -L and -C groups of this study supported this hypothesis. After 12 weeks training, the -L mice fasted for 12 h. At this time point, HSL-Ser563 phosphorylation of VAT in the HIIT-L group increased significantly, which did not happen in the HFD-L or MICT-L groups. This means that with the same fasting period, visceral fat HSL lipolysis in HIIT mice was stronger. To further verify whether higher HSL lipolytic activity is associated with improved catecholamines resistance, visceral and subcutaneous adipocytes of -C groups were isolated after 12 weeks of training without fasting and stimulated by catecholamines of the same physiological concentration. We found that HSL-Ser563 phosphorylation of VAT in the HIIT-C group was significantly higher than in the MICT-C group, while the phosphorylation of SCAT was similar in both groups. The FTIR spectrum data showed that the triglyceride bonds of VAT in HIIT-C groups were lower than in MICT-C groups, indicating that lipolysis was more intense. These data indicated that long-term HIIT improved HFD-induced catecholamines resistance of VAT more than MICT. Better catecholamines sensitivity of VAT means stronger lipolysis under external stimuli, which explains why long-term HIIT reduced more visceral fat than MICT in this experiment. It should be noted that a recent study compared the phosphorylation of visceral fat HSL in a resting state (without fasting) after long-term HIIT and MICT, and found no difference between the two protocols (Maillard et al., 2019). This suggested that long-term HIIT only improves the sensitivity of VAT to catecholamines, rather than HSL lipolytic activity when in a resting state without fasting or exercise. In summary, -L and -C groups showed that visceral fat HSL of long-term HIIT mice is more likely to be activated by fasting or catecholamines stimulation because it causes more TG to be hydrolyzed and more NEFA is released for fat oxidation, gluconeogenesis, or redistribution to skeletal muscle. This phenomenon may be an important mechanism to explain why HIIT can reduce more visceral fat than MICT.

Further, based on the adaptive changes caused by HIIT in this study, two interesting questions could be raised: (1) was the higher muscle TG content of HIIT mice compared to MICT related to the endurance paradox in this study? High levels of intramyocellular TG correlate with insulin resistance in both obese and diabetic patients, but the “good” high muscle TG increases of endurance athletes do not cause insulin resistance, which called “endurance paradox” (Russell, 2004). Is it a “beneficial” change that mice with long-term HIIT gain higher muscle TG content than MICT? and (2) Is HIIT better at preventing obesity rebound? Previous studies have suggested that exercise cessation after MICT appeared to increase adipose accumulation (Del Vecchio et al., 2020), while HIIT showed stronger sustained effects on promoting enzymes activity associated with glycolysis and beta-oxidation pathways than MICT (Gentil et al., 2020). Like other sustained effects, could the better improvement of HIIT on sensitivity of adipocyte lipolysis imply better obese rebound prevention effect? Future studies on these questions may help improve the fat loss theory of HIIT.

### Limitations

Our study had several limitations. Firstly, due to the large workload of a long experimental period (14-week to establish obese mice and 12-week for long-term training) and numerous experimental groups and animals, the normal diet groups were not set to verify whether the effects of HIIT and MICT on improving lipolysis were only seen in obese or HFD-fed mice. Secondly, due to the lower protein content of fat than other tissues, the quantity of proteins that could be extracted after primary adipocyte isolation and stimulation, were not sufficient for HSL protein expression and Ser660 phosphorylation testing, so only Ser563 of adipocytes in -C groups was tested. This choice was based on results of -L groups after fasting, as only Ser563 was significantly different between groups. Finally, it should be noted that there are indeed species-differences in the effect of sympathetic nerve and hypothalamic-adrenal axis on promoting lipolysis (Braun et al., 2018), whether the results of this study are applicable to humans remains to be verified by future studies.

### Conclusion

The present study indicates that an acute HIIT could promote HSL phosphorylation of subcutaneous fat lasting at least 12 h which is longer than MICT, implying HIIT had a longer post-exercise effect on subcutaneous fat lipolysis than MICT. Long-time HIIT was confirmed to have a better effect on improving HFD-induced catecholamines resistance of visceral adipocytes. These effects allow fat to be easier mobilized by fasting, exercise, or other stimulation, which means that more NEFA can be released for energy balance. This study laid the foundation for explaining the mechanism by which HIIT has the same or better weight loss effect than MICT. Future works should explore whether enhanced fat mobilization by HIIT can cause more oxidation, gluconeogenesis, or redistribution of fat to reduce weight.

## Data Availability Statement

All datasets generated for this study are included in the article/supplementary material.

## Ethics Statement

The animal study was reviewed and approved by Ethics Committee of Hebei Normal University.

## Author Contributions

YL and HZ designed the study and analyzed and interpreted the data. YL, GD, XZ, and ZH collected the data. YL drafted the manuscript. HZ and GD revised the manuscript. All authors contributed to the article and approved the submitted version.

### Conflict of Interest

The authors declare that the research was conducted in the absence of any commercial or financial relationships that could be construed as a potential conflict of interest.
